# Effect of Wii Fit Exercise With Balance and Lower Limb Muscle Strength in Older Adults: A Meta-Analysis

**DOI:** 10.3389/fmed.2022.812570

**Published:** 2022-05-06

**Authors:** Haoyan Liu, Yu Xing, Ying Wu

**Affiliations:** Department of Physical Education and Training, Shanghai University of Sport, Shanghai, China

**Keywords:** aged, balance, muscle strength, Wii Fit exercise, meta-analysis

## Abstract

**Background:**

Falls and fall-related injuries are not uncommon among older adults and may lead to serious health deterioration and decreased quality of life. Numerous types of physical activity have been proposed to improve balance and strength in older adults with varying degrees of success. Nintendo's Wii Fit video exergame uses body movement as a game controller and provides real-time feedback for games designed to enhance balance and muscle strength. However, whether Wii Fit exercise improves the balance and lower limb muscle strength of older adults remains uncertain.

**Objective:**

To evaluate the current literature by using meta-analyses to assess whether Wii Fit exercise is associated with improved balance and lower limb muscle strength in older adults.

**Methods:**

We searched PubMed, EMBASE, Web of Science, China National Knowledge Infrastructure, and Wanfang Data from inception to February 16, 2022, for relevant studies and conducted a manual search of the literature. Data extraction of the included studies were independently performed by two authors. The methodologic quality of each included study was assessed using the Physical Therapy Evidence Database (PEDro) scale. Meta-analyses were conducted using Review Manager 5.3.

**Results:**

Of 991 articles initially identified, 10 articles (379 participants; aged ≥65 years, range 65–92 years; 188 participants in Wii Fit exercise groups, and 191 participants in control groups), including 8 randomized controlled trials, were assessed in this meta-analysis. The methodologic quality of the included studies evaluated with the PEDro average score was 6.4 and ranged from 5 to 8 points, indicating moderate quality. Compared with no exercise, Wii Fit exercise was associated with significant improved scores on the Berg Balance Scale, which evaluates functional balance (mean difference, 1.38; 95% CI, 0.61–2.16; *I*^2^ = 31%; *P* = 0.0005) and on the Timed Up and Go test, which evaluates static and dynamic balance (standardized mean difference, −0.51; 95% CI,−0.88 to −0.14; *I*^2^ = 52%; *P* = 0.007). However, for the three studies assessed, Wii Fit exercise did not significantly improve scores on the 30-s chair stand test, which evaluates the functional strength and endurance of the lower limb muscles (mean difference, 0.82; 95% CI, −0.14 to 1.77; *z* = 1.68; *P* = 0.09).

**Conclusions:**

Our findings indicated that Wii Fit exercise is an effective approach to improve functional, static, and dynamic balance among older adults. Additional meta-analyzes with higher numbers of randomized controlled trials are suggested to confirm the benefits of Wii Fit exercise on balance in older adults, to establish whether functional strength and endurance of lower limb muscles are improved, and to explore the relationship between improved balance and fall prevention in this population.

## Introduction

Maintaining balance becomes increasingly difficult with age, as age-related changes occur in sensory systems (vestibular, visual, and somatosensory) and with muscle strength, leading to increased risks of falls and fall-related injuries (1–3). Falling is a significant sign of human aging, with approximately one-third of people older than 65 years injured by falls each year (4). As a common form of injury, falling has high rates of injury and mortality in elderly populations. Fall injury is the second major cause of death associated with accidental or unintentional injury (5). Exercise has a positive effect on balance and helps to prevent falls among older adults (5). However, adherence to traditional sports typically decreases with age among older adults (6). Maintaining balance is a complex process that requires coordination between the sensory system (vision, vestibular, proprioception, etc.) and the motor system (7). Therefore, new approaches for performing exercise that are easy to do, are appealing to older adults to maintain high compliance rates, and that engage multisensory systems and muscle strength are needed to improve balance in this population.

The technology for interactive video games involving physical activity, termed exergames, has evolved and has been applied as an effective approach for treating sarcopenia and cognitive impairment. Video game systems are more cost-effective than developing custom-designed rehabilitation equipment and are becoming increasingly more popular among individuals undergoing physical rehabilitation (8). Among the most common video game systems used for this purpose (Wii Fit, Dance Revolution, EyeToy, and Kinect), Nintendo's Wii Fit is the most popular (9). It was the first video game system to use body movements as a game controller. The exerciser stands on an external balance board and controls the movement of a virtual character in the game by changing the center of pressure on the board. The game provides both visual and auditory feedback to the exerciser. This system combines fun with physical exercise for all ages as it allows for work on joint flexibility, muscular strength, and postural ergonomics. Wii Fit exercise has been shown to be safe for healthy older adults (10). However, the effect of Wii Fit exercise on balance and lower limb muscle strength among older adults is controversial (11, 12). In addition, to our knowledge, no meta-analysis has compared no exercise with the effect of Wii Fit exercise among adults 65 years of age or older. Therefore, the aim of this meta-analysis was to assess the effects of Wii Fit exercise on balance and lower limb strength among seniors aged 65 years or older.

## Methods

Ethical approval was not required for this study because all data were obtained from published articles. This study was reported in accordance with the Preferred Reporting Items for Systematic Reviews and Meta-Analyses (PRISMA) guideline (2009) (13).

### Search Strategy

We performed a systematic search of PubMed, EMBASE, Web of Science, China National Knowledge Infrastructure, and Wanfang Data from their inception to February 16, 2022. Search terms, including “Wii Fit exercise,” “Wii Fit balance board,” “virtual reality,” “exergame,” “interactive,” “video game,” “role-playing,” “real-time strategy,” “balance,” “strength,” “postural control,” “older adults,” “elderly,” “senior,” “older people,” “aged,” “aging,” and “elderly,” were used to retrieve articles. A manual search was also performed.

### Eligibility Criteria

The criteria for a study to be included in the meta-analysis were the following: (1) population: adults aged ≥65 years; (2) intervention: Wii Fit exercise; (3) comparator: Wii Fit exercise vs. non-exercise control; (4) outcomes: studies reporting balance or lower limb muscle strength outcomes; (5) study designs: randomized or non-randomized controlled trials.

Exclusion criteria were as follows: (1) case reports, animal experimental studies, observational studies, reviews, meeting abstracts, and editorials; (2) cognitively or physically limited participants; (3) lack of adequate published information, with authors not providing the requested data; (4) control group with a physical activity intervention.

### Data Extraction

All studies retrieved by our searches were imported into EndNote X9 software, and duplicate studies were removed. First, two independent reviewers (LHY and XY) conducted literature screening. Second, both of these reviewers screened the titles and abstracts and then read the full text of the potentially relevant papers, eliminating irrelevant studies. Third, the two reviewers extracted key information from the included articles. From each included study report, we collected the primary author, country, publication year, age of participants, sample size, characteristics of the Wii Fit exercise program, and outcomes. If there was any disagreement during any portion of the process, the issue was resolved in consensus meetings of the two reviewers.

### Quality Assessment

Two investigators (LHY and XY) independently assessed the risk of bias and quality of the included studies by using the Physical Evidence Database (PEDro) scale. This scale includes 11 items for rating studies. Studies scoring 7 or more points are considered to be of high quality; studies with 5 to 6 points of fair quality, and studies with a score <4 points were excluded from this review. Disagreement was resolved by consensus with a third investigator (WY).

### Statistical Analysis

Meta-analyses were performed using Review Manager 5.3 software (Cochrane, UK). Data from the included studies were used to estimate the standardized mean difference (SMD) or mean difference (MD). Heterogeneity was calculated using the *I*^2^ statistic. *I*^2^ statistic values of 25–50, 50–75, and 75–100% represented low-, moderate-, and high-level heterogeneity, respectively. When *I*^2^ ≥ 50% or *P* < 0.10, sources of heterogeneity were explored by a subsequent subgroup analysis. A random-effects model was used for *I*^2^ < 50%, and a fixed-effects model was used when *I*^2^ ≥ 50%. Egger's test were used to assess publication bias by Stata 1.4. Values of *P* < 0.05 were considered to be statistically significant. Medians and interquartile ranges (IQRs) in studies were replaced by medians with standard deviations (SDs) using the formula SD = IQR/1.35 (14).

## Results

### Search Results

**Figure 1:**
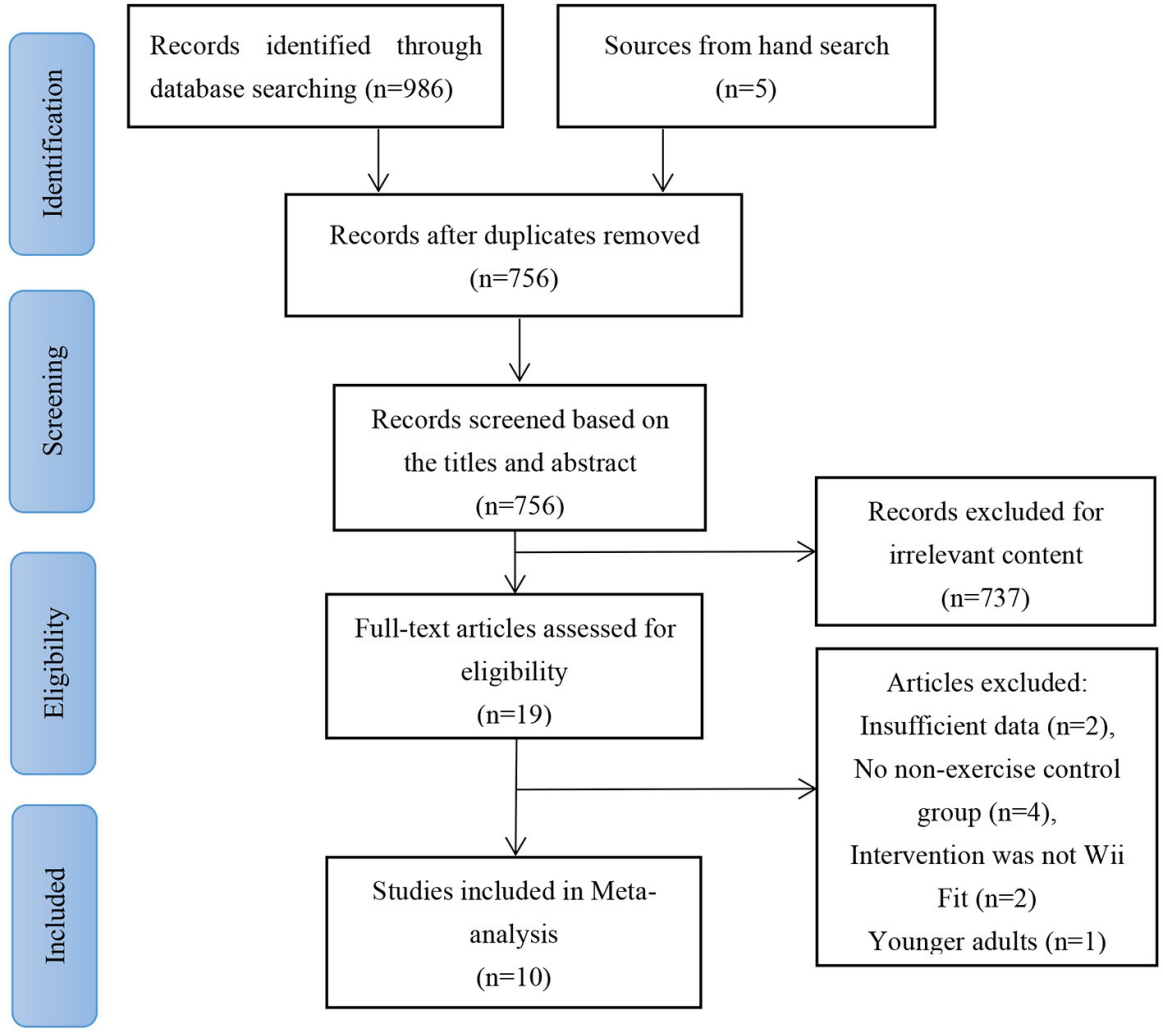
PRISMA flowchart.

The selection process using the inclusion/exclusion criteria and the flowchart of study selection is given in Figure 1. A total of 991 potentially relevant articles were identified. After the screening process, 235 duplicate articles were removed by EndNote X9. In total, 737 irrelevant articles were removed through the title and abstract screening, 19 full-text articles were read, and 9 articles excluded. Thus, a total of 10 articles (12, 15–23) were selected for the meta-analysis.

### Study Characteristics

The study characteristics are presented in Table 1. Of 10 included studies, 8 were randomized controlled trials (12, 15–20, 23), and 2 were non-randomized controlled trials with similar baselines (21, 22). The studies were conducted in 6 countries, including the US (*n* = 4), Korea (*n* = 2), Australia (*n* = 1), the UK (*n* = 1), Denmark (*n* = 1), and Lebanon (*n* = 1). Among the 10 included studies, 188 older adults participated in the Wii Fit exercise groups, and 191 older adults participated in the control groups. The ages of the participants ranged from 65 to 92 years. All studies compared Wii Fit exercise with no exercise.

**Table 1:** Baseline characteristics of included studies.

**References**	**Region**	**Sample No. (Wii Fit/control)**	**Age (mean ±SD or range, years)**	**Intervention**				**Adherence (%)**	**Outcomes**
				**Frequency (time/week)**	**Duration (weeks)**	**Time (min)**	**Games (n)**		
Bieryla et al. (12)	United States	5/5	81.5 ± 5.5	3	3	30	6	83.3	BBS; TUG
Chao et al. (15)	United States	16/16	85.19 ± 6.47	2	4	30	6	100	BBS; TUG
Fakhro et al. (16)	Lebanon	30/30	72.2 ± 5.2	3	8	30	2	90.9	TUG
Franco et al. (17)	United States	11/10	78.27 ± 6	2	3	13	5	100	BBS
Jorgensen et al. (18)	Denmark	28/30	75 ± 6	2	10	35	6	100	TUG; 30-s chair stand
Lee et al. (19)	Korea	9/8	69–82	2	8	30	9	75	TUG
Lee et al. (20)	Korea	21/19	71–81	2	6	60	6	95.4	BBS; TUG
Nicholson et al. (21)	Australia	19/22	65–84	3	6	30	8	100	TUG; 30-s chair stand
Orsega-Smith et al. (22)	United States	9/9	71.6 ± 7.79	2	8	30	10	100	BBS; TUG; 30-s chair stand
Whyatt et al. (23)	United Kingdom	40/42	69–84	2	5	30	4	95.2	BBS

*BBS, Berg Balance Scale; TUG, Timed Up and Go test; SD, standard deviation*.

### Risk of Bias

The results of the quality assessments conducted using the PEDro scale are shown in Table 2. The average score was 6.4 points, and the quality of studies ranged from 5 to 8 points, with five studies scoring 7 or higher points (12, 16, 18, 20, 23), indicating that the quality of the included studies was moderate.

**Table 2:** Evaluation using the Physiotherapy Evidence Database (PEDro) scale for the level of evidence of all included studies.

**References**	**Eligibility criteria specified**	**Random allocation**	**Concealed allocation**	**Baseline comparability**	**Blinded participants**	**Blinded therapists**	**Blinded assessors**	**Adequate follow-up**	**Intention-to-treat analysis**	**Between-group comparison**	**Point estimates and variability**	**PEDro Score**
Bieryla et al. (12)	1	1	0	1	0	0	0	0	0	1	1	5
Chao et al. (15)	1	1	1	1	0	0	0	1	1	1	1	8
Fakhro et al. (16)	1	1	0	1	0	0	0	1	0	1	1	6
Franco et al. (17)	1	1	0	1	0	0	0	1	1	1	1	7
Jorgensen et al. (18)	1	1	1	1	0	0	0	1	0	1	1	7
Lee et al. (19)	1	1	0	1	0	0	0	1	0	1	1	6
Lee et al. (20)	1	1	1	1	0	0	0	1	0	1	1	7
Nicholsonet al. (21)	1	0	0	1	0	0	0	1	0	1	1	5
Orsega-Smith et al. (22)	1	0	0	1	0	0	0	1	1	1	1	6
Whyatt et al. (23)	1	1	0	1	0	0	0	1	1	1	1	7

### Meta-Analysis

#### Berg Balance Scale Scores

**Figure 2:**
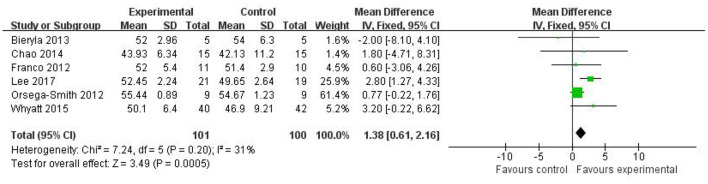
Effect of Wii Fit with berg balance scale.

The BBS score is one of the most common measurements for evaluating functional balance in elderly populations. Six of 10 studies (12, 15, 16, 20, 22, 23) involving 201 elderly participants were included in the analysis of functional balance. The results of the meta-analysis suggested that the Wii Fit exercise program significantly improved functional balance (MD = 1.38, 95% CI, 0.61–2.16; *I*^2^ = 31%; *P* = 0.0005), with low-level heterogeneity and using a fix-effects model (Figure 2).

#### Time Up and Go Test

**Figure 3:**
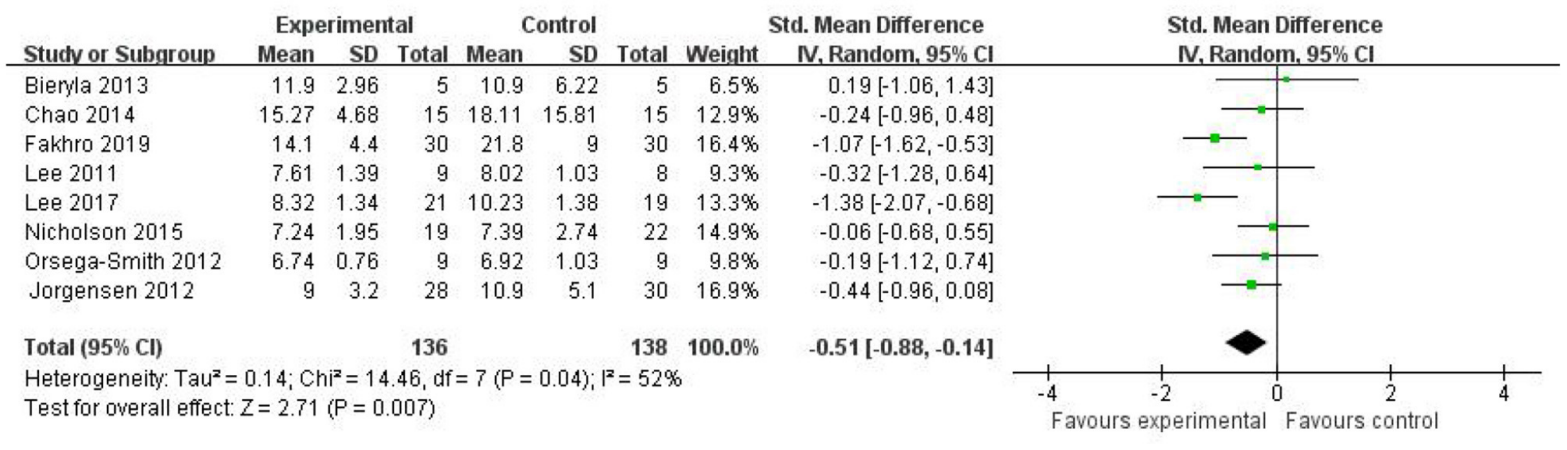
Effect of Wii Fit exercise with timed up and go test.

The TUG test is an effective test to evaluate static and dynamic balance in older adults. Less time to complete the TUG test indicates better balance. The meta-analysis pooled from eight studies (15–23) showed that there was a statistically significant difference in TUG scores between the Wii Fit group and the control group, with the Wii Fit group spent significantly less time completing the TUG (SMD = −0.51; 95% CI, −0.88 to −0.14; *I*^2^ = 52%; *P* = 0.007), with moderate-level heterogeneity and using a random-effects model (Figure 3).

#### Thirty-Second Chair Stand Test

**Figure 4:**
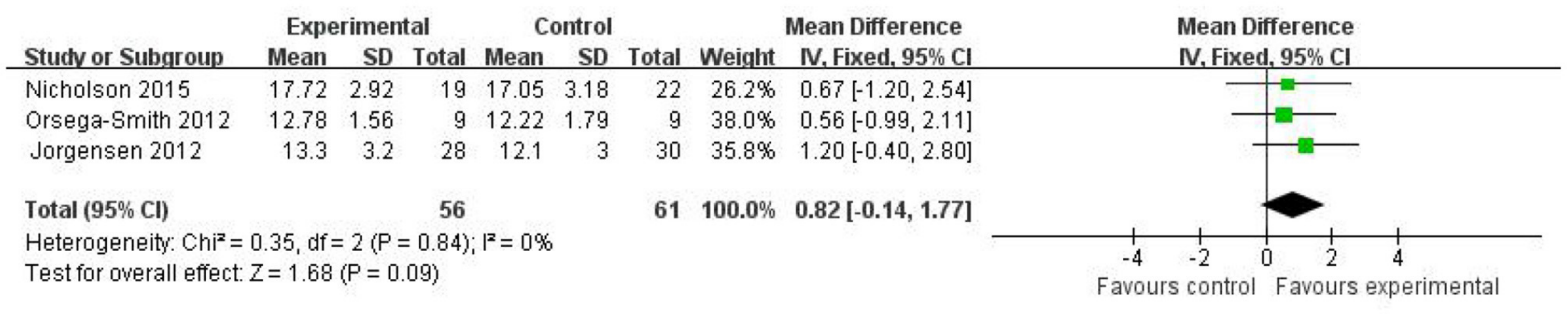
Effect of Wii Fit exercise with 30-s chair stand test.

The 30-second chair stand test evaluates the level of functional strength and endurance in the lower limb muscles. Three (18, 21, 22) studies were included in the meta-analysis to determine the effects of Wii Fit exercise on lower limb muscle strength compared with a non-exercise control group (Figure 4). The results of the meta-analysis suggested that participants in the Wii Fit exercise program showed improvement in lower limb muscle strength; however, the pooled results showed that strength was not statistically significantly different between the Wii Fit exercise and control groups (MD = 0.82; 95% CI, −0.14 to 1.77; *z* = 1.68, *P* = 0.09), with homogeneity and using a fix-effects model.

### Sensitivity Analysis

The results of subgroup analyses suggested that the heterogeneity for two outcomes was reduced (*I*^2^ = 0% for exercise frequency and number of exercise weeks). Thus, exercise frequency and number of weeks that exercise was performed may be sources of heterogeneity (Supplementary Table 3). Excluding one article at a time to assess the influence of a single study on this meta-analysis showed that overall, heterogeneity was not changed and that the conclusions were consistent with the main analyses.

### Publication Bias

The results of the Egger's test suggested that there were no significant publication biases in the meta-analyses that included the three measures assessed herein: the Berg Balance Scale (BBS) (*P* = 0.898), the Timed Up and Go (TUG) (*P* = 0.364), and the 30-s chair stand tests (*P* = 0.850).

## Discussion

The results of this meta-analysis indicated that Wii Fit exercise may be an effective strategy to improve functional, static, and dynamic balance in older adults. Compared with older adults who did not exercise, those who participated in Wii Fit exercise showed substantial improvement in the BBS and TUG test scores. However, this meta-analysis failed to observe a significant impact of Wii Fit exercise on lower limb muscle strength, which may be attributable to the small number of evaluated studies (*n* = 3) and adopt different games. Evidence from similar systematic reviews assessing other populations are in agreement with our findings of improved balance through Wii Fit exercise (24–26). For example, a recent meta-analysis concluded that compared with controls, older adults with neurocognitive disorders who performed Wii Fit exercise significantly improved their BBS test scores (25). That study assessed the methodologic quality of studies with the GRADE assessment tool and reported that three of five studies had high risk of bias. Another review that assessed the effect of Wii Fit on BBS scores found that Wii Fit exercise improved BBS scores compared with traditional therapy (26). Unlike our study, that meta-analysis included participants aged 12–86 years as well as participants with various medical conditions, such as Alzheimer's disease, Parkinson's disease, and stroke. Because of the diverse ages and medical conditions, the results of that study should be cautiously interpreted.

The efficacy of Wii Fit exercise to improve balance among older adults remains controversial. Iruthayarajah et al. suggested that Wii Fit exercise was not associated with improved BBS and TUG test scores among individuals with chronic stroke compared with conventional rehabilitation (27). Similarly, a systematic review suggested that Wii Fit training may not improve dynamic balance because Wii Fit exercise uses only stepping actions inside the base of support, and this type of exercise may not improve TUG test scores (28). However, another study stated that most Wii Fit games require participants to shift their weight to the left and right to achieve success, and this type of exercise may improve medial-lateral stability. Improvements in medial-lateral stability may be associated with positive changes in dynamic balance (21). In our meta-analysis, Wii Fit exercise was associated with improved BBS and TUG test scores compared with scores in the non-exercising control group. Our meta-analysis included healthy older adults aged ≥65 years, whereas the study by Iruthayarajah et al. included adults with chronic stroke aged >19 years. These differences likely contributed to the disparate outcomes between the studies.

Wii Fit exercise provides a sensory-enriched environment for individuals as they adjust their own balance and movements according to visual, auditory, and proprioceptive feedback while performing an exercise (29, 30). This stimulates their vestibular system and proprioceptors and enables exercisers to focus on the mobility required to play the game. A previous study stated that Wii Fit exercisers showed significant improvements in their vestibular integration and visual integration abilities compared with the control group immediately after the training as well as at the 1-month follow-up (31). In addition, impaired lower limb muscle strength is one of the primary factors for fall accidents in elderly (32). Our analyses suggest Wii Fit exercise may be promote adaptations in muscle strength, but do not have significantly different. A randomized controlled trial examined 10 weeks of Wii Fit exercise on lower limb muscle strength in older community-dwelling adults and found a significant improvement, with an increase of ~20% in maximal muscle strength and in rapid force generation (18). The results of another randomized controlled trial suggested that the strength of the hip, knee, and ankle joints of the participants was significantly improved through the Wii Fit exercise and that the effect of the Wii Fit exercise in improving maximal isometric muscle strength in older adults was similar to that of traditional exercise training (31). In the Strength-enhancing Wii Fit exercise study, these protocol involved strength training games. Such as table tilt and slalom ski (18, 31, 33). Future research could explore the effects of different game programs on muscle strength in older adults. In terms of cognitive function, a previous study assessed the effects of Wii Fit exercise on cognitive function among stroke patients and observed significant benefits of interactive physical activity video-game training on cognitive function (34). Wii Fit exercise had significant effect in cognitive function as compared to Tetrax balance system. In short, Wii Fit exercise games may improve balance in older adults by promoting sensory system functions and enhancing muscle strength and cognitive function.

The present study found high adherence (100%) to Wii Fit exercise among older adults in five of 10 Wii Fit exercise studies. A recent meta-analysis demonstrated that Wii Fit exercise was safe, with no adverse events and high adherence (>80%) and low attrition (<20%) rates in four of five Wii Fit exercise studies (25). Kirk et al. stated that participants reported that Wii Fit exercise was easier to perform and the games was more amusing and engaging compared with traditional balance training. Wii Fit exercise improved participants' enjoyment and motivated them and increased their desire to participate (35), as Wii Fit exergame provides interesting images and music. The system contains a large number of sports games to choose from, allowing older adults to participate in personalized and interesting tasks. Real-time visual and auditory feedback enables the participant to be more interested in Wii Fit exercise and helps to ensure that older adults continue to participate in Wii Fit exercise (18). Moreover, Wii Fit exercise provides opportunities for social interaction with friends and family members, and the cost of Wii Fit video game system is as low as US $250 (15). All those features make Wii Fit exercise attractive to older adults.

## Limitations

There several limitations to this study. First, few studies were included (*n* = 10), two of which were not randomized controlled trials although those two studies reported similar baselines. This small number highlights the need for more randomized controlled trials in general and to confirm our findings in particular. Second, few studies have used an exercise control group when evaluating the effect of Wii Fit exercise in healthy older adults. Third, the generalizability of our results should be considered because the included studies (1) were published only in English and Chinese languages, and (2) recruited only adults ≥65 years of ages. Thus, our findings may not be generalizable to other populations.

## Conclusions

In this meta-analysis, Wii Fit exercise was shown to improve functional, static, and dynamic balance in older adults. Although not statistically significant, perhaps owing to the small sample size, this type of exercise was also associated with enhanced lower limb muscle strength. Future meta-analyses should confirm our results by assessing a larger sample of randomized controlled trials. Future research directions should include exploration of the effect of Wii Fit exercise on the relationship between muscle strength and different games among older adults.

## Data Availability Statement

The original contributions presented in the study are included in the article/Supplementary Material, further inquiries can be directed to the corresponding author/s.

## Author Contributions

YL and YW designed the research study. YL and YX conducted the search strategy, conducted study screening and data extraction, and wrote the first draft of the paper. YL analyzed the data. YL, YX, and YW interpreted the data. All authors have read and approved the final manuscript.

## Conflict of Interest

The authors declare that the research was conducted in the absence of any commercial or financial relationships that could be construed as a potential conflict of interest.

## Publisher's Note

All claims expressed in this article are solely those of the authors and do not necessarily represent those of their affiliated organizations, or those of the publisher, the editors and the reviewers. Any product that may be evaluated in this article, or claim that may be made by its manufacturer, is not guaranteed or endorsed by the publisher.
