# Supplementary figures and images for: Effect of Wii Fit Exercise With Balance and Lower Limb Muscle Strength in Older Adults: A Meta-Analysis
Source: Front Med (Lausanne). 2022 May 6;9:812570. doi: 10.3389/fmed.2022.812570 (PMC9120538; doi:10.3389/fmed.2022.812570)

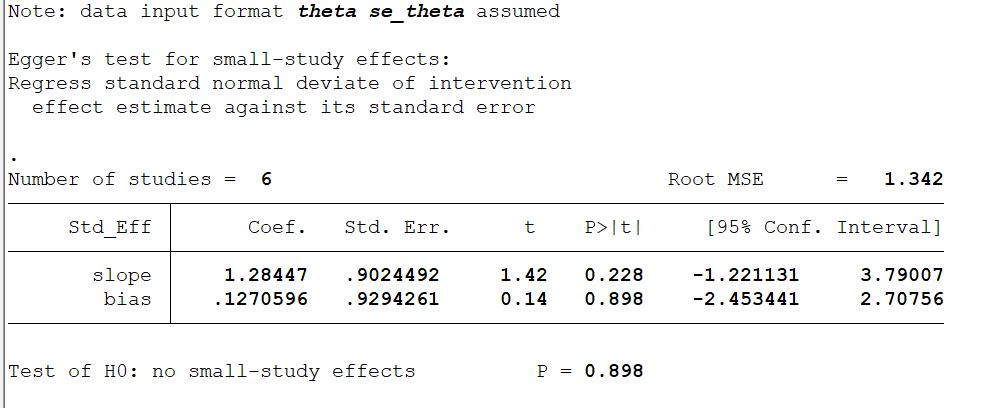


BBS


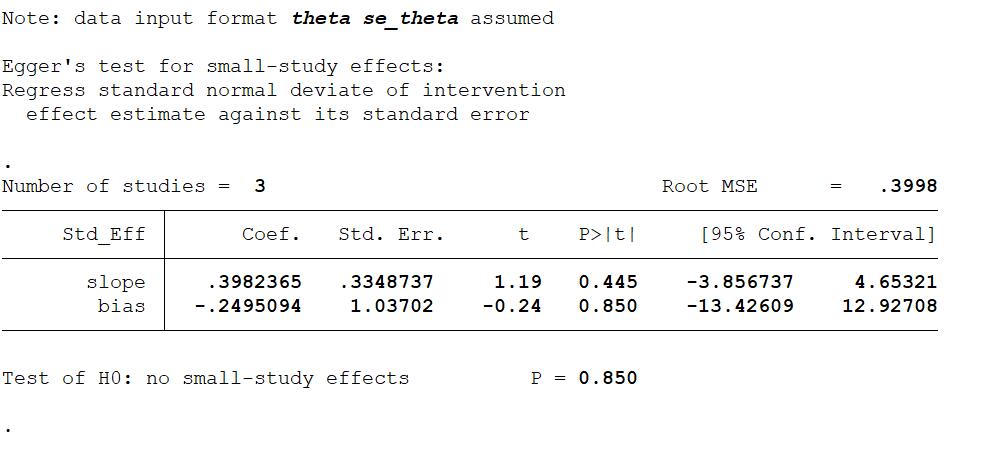


TUG test


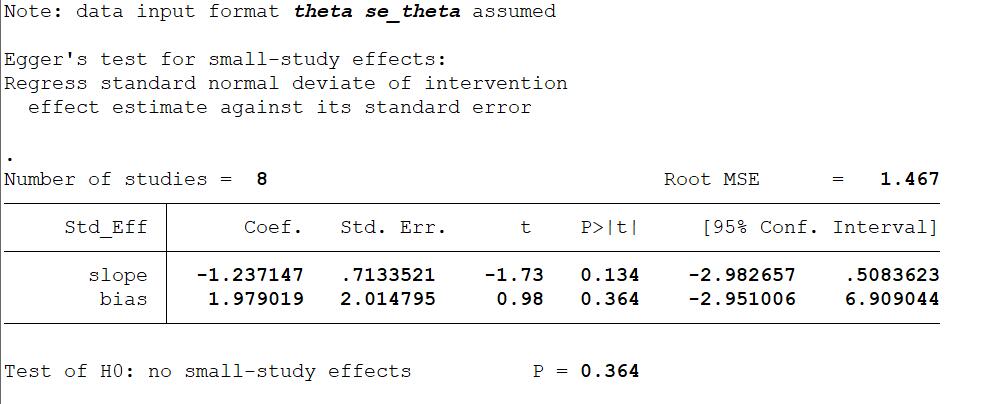


30-Second Chair Stand Test

Supplement: Supplementary file 1 [file Data_Sheet_1.docx]
