# Supplementary material for: Effect of Wii Fit Exercise With Balance and Lower Limb Muscle Strength in Older Adults: A Meta-Analysis
Source: Front Med (Lausanne). 2022 May 6;9:812570. doi: 10.3389/fmed.2022.812570 (PMC9120538; doi:10.3389/fmed.2022.812570)
Supplement: Supplementary file 2 [file Table_3.DOCX]

Supplementary TABLE 3 | Subgroup analysis of the effect of Wii Fit exercise on TUG of older adults.

|  | Subgroup | K(sample) | ES (95%CI) | P | I²/% |
| --- | --- | --- | --- | --- | --- |
| Age  (years) | 65~80 | 6（234） | -1.04，-0.18 | 0.006 | 59 |
|  | 80~95 | 2（40） | -0.75，0.49 | 0.68 | 0 |
|  | total | 8（274） | -0.88，-0.14 | 0.007 | 33.9 |
| Duration  (weeks) | ≤6 | 3（121） | -1.12，0.27 | 0.23 | 69 |
|  | ＞6 | 5（153） | -1.01，-0.18 | 0.005 | 31 |
|  | total | 8（274） | -0.88，-0.14 | 0.007 | 0 |
| Frequency  (time/week) | 2 | 5（163） | -0.98，-0.10 | 0.02 | 44 |
|  | 3 | 3（111） | -1.22，0.41 | 0.33 | 73 |
|  | total | 8（274） | -0.88，-0.14 | 0.007 | 0 |
| Time  (min) | 13-30 | 6（176） | -0.74，-0.13 | 0.005 | 40 |
|  | 35-60 | 2（98） | -1.19，-0.36 | 0.0003 | 78 |
|  | total | 8（274） | -0.80，-0.30 | 0.007 | 40.9 |
| Games  (n) | 2~6 | 5（198） | -1.16，-0.19 | 0.006 | 60 |
|  | 8~10 | 3（76） | -0.60，0.30 | 0.52 | 0 |
|  | total | 8（274） | -0.88，-0.14 | 0.007 | 59.2 |

*TUG:Time Up and Go Test;K: number of studies;CI: confidence interval;P: p-value;ES: effect size*
